# Beta-Glucans in Biotechnology: A Holistic Review with a Special Focus on Yeast

**DOI:** 10.3390/bioengineering12040365

**Published:** 2025-03-31

**Authors:** Nirmal Sarkar, Atharva Anand Mahajan, Sagarjyoti Pathak, Prakriti Seth, Ankita Chowdhury, Indrilla Ghose, Shrimanti Das, Rajanyaa Chowdhury, Aishi Bera, Anuvab Dey, Anushka Dutta, Ipsita Majumder, Subhrojyoti Ghosh, Ramya Lakshmi Rajendran, Prakash Gangadaran

**Affiliations:** 1 Department of Biosciences and Bioengineering, Indian Institute of Technology Guwahati, North Guwahati 781039, Assam, Indiaspathak18dgp13@gmail.com (S.P.); anuvab2000dey@gmail.com (A.D.); 2 Advance Centre for Treatment, Research and Education in Cancer (ACTREC), Tata Memorial Centre, Navi Mumbai 410210, Maharashtra, India; atharva66mahajan@gmail.com; 3 Department of Biotechnology, National Institute of Technology Raurkela, Sector 1, Rourkela 769008, Odisha, India; prakritiseth16@gmail.com; 4 Department of Biochemical Engineering and Biotechnology, Indian Institute of Technology Delhi, New Delhi 110016, Delhi, India; chowdhuryankita05@gmail.com; 5 Department of Chemistry and Chemical Biology, Indian Institute of Technology (ISM), Dhanbad 826004, Jharkhand, India; indrillaghose.01@gmail.com; 6 Department of Biotechnology, Heritage Institute of Technology, Kolkata 700107, West Bengal, India; shrimantidas1210@gmail.com (S.D.); rajanyaa.chowdhury.bt23@heritageit.edu.in (R.C.); anushkadutta2002@gmail.com (A.D.); ipsitamajumder@gmail.com (I.M.); 7 Department of Bioengineering and Biomedical Engineering, University of Florida, Gainesville, FL 32611, USA; aishib510@gmail.com; 8 Department of Biotechnology, Indian Institute of Technology Madras, Chennai 600036, Tamil Nadu, India; subhrojyotighosh8@gmail.com; 9 BK21 FOUR KNU Convergence Educational Program of Biomedical Sciences for Creative Future Talents, Department of Biomedical Sciences, School of Medicine, Kyungpook National University, Daegu 41944, Republic of Korea; 10 Department of Nuclear Medicine, School of Medicine, Kyungpook National University, Daegu 41944, Republic of Korea; 11 Cardiovascular Research Institute, Kyungpook National University, Daegu 41944, Republic of Korea

**Keywords:** beta-glucans, yeast fermentation, immunomodulation, health benefits, biomedical applications, *Saccharomyces cerevisiae*, polysaccharides, biotechnological uses

## Abstract

Beta-glucans (β-glucans) are polysaccharides with significant biological activity, widely recognized for their immunomodulatory, anti-inflammatory, and metabolic health benefits. Among various sources, yeast-derived β-(1 → 3), (1 → 6)-glucans have gained particular attention due to their potent bioactivity and diverse applications in biotechnology, pharmaceuticals, and functional foods. This review comprehensively examines yeast β-glucans, covering their biosynthesis, extraction, and purification from industrially relevant yeast strains, particularly *Saccharomyces cerevisiae.* The impact of fermentation parameters on β-glucan yield and structural properties is analyzed, highlighting advancements in optimizing microbial production. Furthermore, we discuss methods for characterizing yeast β-glucans, including analytical and bioassay techniques, and compare their physicochemical properties with those of β-glucans from other sources. Finally, this review explores the therapeutic potential of yeast-derived β-glucans, focusing on their role in immunomodulation and metabolic regulation and their emerging applications in biomedicine, functional foods, and industrial formulations. By synthesizing recent advancements, this work provides insights into the expanding utilization of yeast β-glucans and their potential for future biotechnological developments.

## 1. Introduction

β-glucans are large polysaccharides of D-glucose monomers connected through β-glycosidic bonds. They are prevalent in nature and can be found in diverse sources, including yeast, mushrooms, bacteria, algae, barley, and oats [1]. These versatile compounds demonstrate various biological activities, including anti-tumor, immune-modulating [2], anti-aging, and anti-inflammatory properties. One of the reasons for the continued interest in β-glucans lies in the diversity of their physical and chemical properties, which can vary depending on the source and molecular weight [3]. For instance, β-glucans from baker’s yeast comprise β-(1 → 3) and (1 → 6) linkages, while β-glucans from cereals are characterized by β-(1 → 3) and β-(1 → 4) linkages. Other variations include cellulose, a (1 → 4) β-glucan, curdlan, a (1 → 3) β-glucan, and lichen-derived β-glucan, which may contain (1 → 3), (1 → 4), or (1 → 3) (1 → 6)-β-glucans. These structure variations contribute to their unique bioactivities, with fungal β-glucans acting as immune system boosters and anti-tumor agents, while β-glucans from cereals aid in lowering cholesterol and blood glucose levels [1].

Additionally, β-glucans can be categorized as soluble and insoluble forms, with their solubility influenced by the degree of polymerization (DP). β-glucans with DPs greater than 100 are typically insoluble in water [3]. These β-glucans find applications in daily life, including beverage thickeners and food additives in products like milk, yogurt, and bread for calorie reduction and cholesterol management [4]. Furthermore, β-glucans are utilized as functional ingredients in producing nutritional and healthy products and are even incorporated into pharmaceutical formulas for supplementary functions. Additionally, their moisturizing, anti-aging, and wound-healing properties make β-glucans valuable components in cosmetics [3].

Recognized for their numerous health benefits, such as immunomodulation, anti-tumor activity, serum cholesterol and glucose reduction, and obesity prevention, β-glucans have a significant presence in the healthy food and pharmaceutical industries. Indeed, according to the Food Marketing Institute (FMI), health and dietary supplement products containing β-glucans accounted for a significant 33.7% of the global β-glucans market share in 2014. Cereal-derived β-glucans remain the dominant source, with yeast- and mushroom-derived β-glucans closely behind. Extensive research has been conducted on various aspects of β-glucans, including production and industrial applications, characteristics [1], and modification [5,6]. This comprehensive review delves into the intricate world of β-glucans produced by yeasts, from fermentation to diverse applications.

## 2. Types of Yeasts Used in Beta-Glucan Fermentation

**Figure 1:**
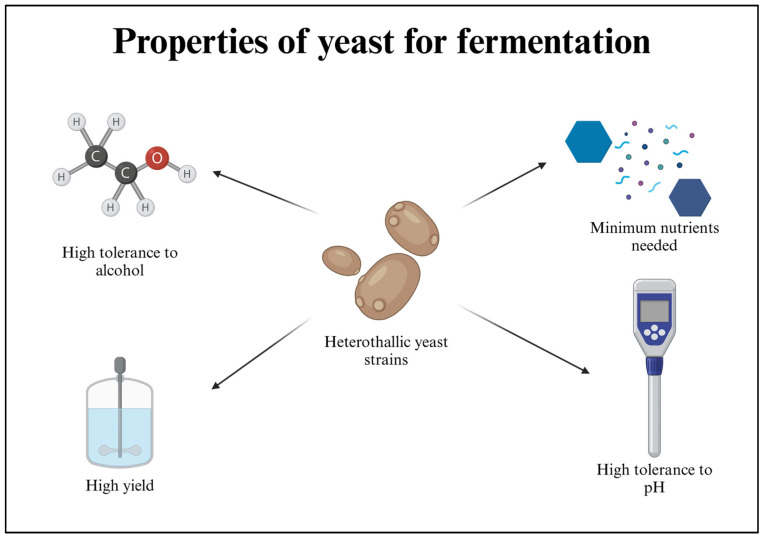
Properties that yeast should have for fermentation: Yeast should have a high tolerance to alcohol to produce a high amount, which will not be toxic for the yeast itself. It should grow in minimal and cost-effective nutrients. It should have a high production capacity and be tolerant of minor changes in the pH, as it can affect the yield.

During the process of brewing in beer production, yeasts are found to make use of fermentable sugars for the production of ethanol and carbon dioxide, during which some by-products are generated, which includes brewer’s spent yeast (BSY), which is known to have high contents of carbohydrate and protein [7,8,9]. Although, by and large, BSY is either inappropriately discarded or utilized as animal feed [10,11], given that it has a rich composition, it can be used as a source of macro-compounds like β-glucan [12,13,14] if one can overcome the added difficulty in isolation and recovery of the compound [12]. β-glucans, one of the polysaccharides in the yeast cell wall attached to the mannoprotein complex, give structural support to the cell [15,16]. *Saccharomyces cerevisiae* is the most widely used yeast in industrial fermentation processes. In recent years, *Brettanomyces bruxellensis*, a distant relative of *S. cerevisiae*, has also been used. However, there is currently a lack of sufficient data and proof on the quantitative number of β-glucans present in the aforementioned species, which demands further research. Figure 1 demonstrates the essential properties that yeasts should have for fermentation.

## 3. Fermentation Process for Beta-Glucan Production

### 3.1. Substrate Selection

Choosing the right substrate is crucial, as the properties of β-glucans vary depending on the source. Differences in viscosity, solubility, and molecular mass result in variations in β-glucans obtained from various sources [17]. For instance, bacterial sources secrete β-glucan in the media, while in yeasts and fungi, it forms a cell wall component. *Xanthomonas campertis*, a Gram-negative bacterium, produces β-glucan in its cyclic form, which is widely used in the food, oil, and cosmetics industries [18]. The cyclic forms of β-glucan can also help encapsulate several compounds like curcumin, reserpine, etc. [19]. *Bacillus subtilis*, a Gram-positive bacterium, also produces β-glucan as a component of its cell wall but in relatively smaller amounts [20]. The famous yeast *S. cerevisiae* produces a large amount of β-glucan, as it is a significant component of its cell wall (about 80 to 90%). This form of β-glucan is considered one of the purest forms and is mainly used in the food and pharmaceutical industries. It is also the most economical form of β-glucan because of the low level of purification required [21,22].

### 3.2. Inoculum Development

While many microorganisms can produce β-glucan, few are efficient and quick. Additionally, some of these microorganisms can threaten the well-being of humans, animals, and plants. To mitigate this issue, researchers have conducted studies to improve the production of β-glucans by developing the strains. Chemical mutagenesis is one such method that can help in inoculum development. This can be achieved by treating the strain with a solution of N-methyl-N-nitro-nitrosoguanidine, as was performed in the case of *Agrobacterium* sp. [23]. Another method involves physical mutation, like exposure to UV light. It can lead to a threefold increase in β-glucan production compared to the wild strain. Apart from the increase in production, the β-glucans also showed low melanin content, which can lead to higher glucan recovery [24].

### 3.3. Fermentation Conditions and Parameters

Optimum β-glucan production depends on several conditions like (i) temperature, pH, fermentation time, and other physical parameters; (ii) optimum medium composition that includes carbon, nitrogen, vitamins, and other micronutrients; and (iii) different methods of fermentation. The most significant factor in the group of physical parameters is the fermentation temperature. The optimum temperature range for maximum yield is from 25 °C to 35 °C. Temperatures from this range have shown reduced reproduction rates and increased pathogenicity in microbes producing β-glucan [25]. The medium’s pH and fermentation’s time duration also significantly affect optimum production. pH values are directly proportional to the yield of β-glucans but inversely related to their molecular weight. The optimum pH range is 3 to 6.5, but some fungal strains have also shown higher yields in the alkaline range [26]. Furthermore, the late exponential growth phase and stationary stage show maximum β-glucan yield, thus proving the contribution of fermentation time in the process as well [27].

Coming to the factor of medium composition, macronutrients like carbon and nitrogen, as well as micronutrients like vitamins and additives, play an equally important role. Being readily available and affordable, simple sugars like glucose, sucrose, and maltose are the most used carbon sources for β-glucan production. Corn starch, potatoes, and sugarcane molasses have also been studied as substrates, especially in fungal strains. However, concentrations of carbohydrates also play a significant role in production. High carbohydrate concentrations can reduce the yield due to induced osmotic stress. Thus, 3% to 6% of carbon substrate concentration is optimum [28]. Nitrogen sources based on ammonium salts have demonstrated enhanced β-glucan production when compared to alternative salts. Lower nitrogen concentrations have been observed to promote β-glucan production, whereas higher concentrations may have inhibitory effects. Effective beta-glucan production has been noted within nitrogen concentrations from 0.1% to 1% [29]. Since the microbes responsible for producing β-glucans are mainly aerobic or facultative aerobes, shake flasks produce an optimum fermentation environment compared to most bioreactors, as they can provide the best constant aeration. Air-lift bioreactors are preferred for industrial production due to their optimum aeration capacity [30].

## 4. Methods for Screening Beta-Glucans

### 4.1. Chemical Assays for Beta-Glucan Detection

Lugol’s Iodine staining, also known as iodine–potassium iodide staining, is a classic and straightforward technique commonly employed to detect β-glucans and other polysaccharides. The method is based on the chemical reaction between iodine and the polysaccharide structure of β-glucans, which results in a color change that can be visually observed. When Lugol’s Iodine solution, which consists of iodine and potassium iodide dissolved in water, is applied to a sample, the iodine molecules interact with the β-glucan molecules. This interaction forms a reversible inclusion complex that gives rise to a deep blue or purple coloration. While Lugol’s Iodine staining is qualitative and cannot precisely quantify β-glucans, it is still highly valuable for several reasons. First, it is a rapid and easily accessible technique, making it ideal for quick preliminary assessments in various fields, such as microbiology and mycology. It is commonly used to identify the presence of β-glucans in fungal cell walls, where these polysaccharides play a significant role in the structural integrity of the organism. The color change, ranging from yellow to brown to blue or purple, indicates the presence and distribution of β-glucans within the sample.

The phenol–sulfuric acid method, commonly called the phenol–sulfuric acid assay, is a well-established and versatile technique for detecting and quantifying β-glucans. It relies on the fundamental chemistry of carbohydrates, particularly their reaction to sulfuric acid. In this assay, a sample suspected of containing β-glucans is mixed with concentrated sulfuric acid and a phenol solution. The acid hydrolyzes the β-glucans into their constituent monosaccharides, primarily glucose. The hydrolysis reaction is exothermic, releasing heat and leading to the formation of complex reaction products. In the presence of phenol, these products undergo further chemical reactions that result in the generation of colorful compounds, which are measurable using a spectrophotometer. The intensity of the color formed is directly correlated with the concentration of β-glucans in the sample. This makes it possible to estimate the amount of β-glucans present. The assay can be calibrated using known concentrations of β-glucan standards to create a standard curve for more accurate quantification. The phenol–sulfuric acid method is known for its simplicity, cost-effectiveness, and speed, making it a valuable initial screening tool [31].

### 4.2. Microscopic Examination

Microscopic examination of β-glucans is a fundamental technique that allows researchers to delve into the structural intricacies of these polysaccharides. β-glucans are widely distributed in nature and found in diverse biological sources such as fungi, bacteria, and grains, making their study an essential aspect of various scientific disciplines, including microbiology, food science, and pharmaceutical research. Light microscopes, often called optical or compound microscopes, are commonly used for initial observations of β-glucan-containing samples. These microscopes are invaluable for providing an overview of the physical characteristics of β-glucans. Researchers can observe these polysaccharides’ size, shape, and distribution within the sample. β-glucans can vary significantly in appearance, from elongated or irregularly shaped structures to different colors, depending on their source and surrounding matrix. When a more detailed analysis of β-glucan structure is required, advanced microscopes like transmission electron microscopes (TEMs) and scanning electron microscopes (SEMs) come into play. TEMs offer high-resolution images, allowing researchers to explore the internal structure of β-glucans, including their layering and arrangement. On the other hand, SEMs provide detailed information about the surface morphology, revealing how β-glucans interact with other components in the sample. In some cases, staining techniques may be employed to enhance contrast, making it easier to distinguish β-glucans from other sample constituents. Stains such as calcofluor white and Congo red are commonly used to highlight β-glucans under the microscope selectively. Microscopic examination is not only beneficial for fundamental research but also for practical applications. In the food industry, for instance, it helps ensure the quality and distribution of β-glucans in products like oat-based cereals and bakery items. In pharmaceutical research, it aids in understanding the bioavailability and drug delivery potential of β-glucans [32].

### 4.3. Modern Screening Techniques

**Figure 2:**
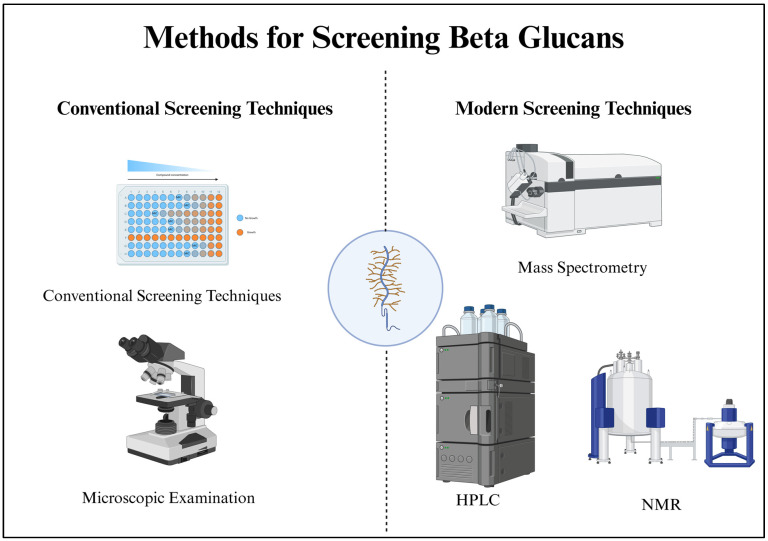
Overview of methods for screening β-glucans. Screening approaches are categorized into conventional and modern techniques. Conventional methods include chemical assays and microscopic detection. At the same time, modern techniques involve advanced analytical tools such as mass spectrometry (MS), nuclear magnetic resonance (NMR), and high-performance liquid chromatography (HPLC) for improved accuracy and sensitivity.

Modern screening techniques for β-glucans represent a significant leap in the capabilities of researchers and industries to understand, quantify, and utilize these biologically and industrially essential polysaccharides. Enzymatic assays, for instance, offer precise quantification through the action of specific enzymes that break down β-glucans into measurable products. High-performance liquid chromatography (HPLC) has become a cornerstone of β-glucan analysis, providing quantification and the ability to distinguish between different β-glucan types, such as those found in oats or fungi. Nuclear magnetic resonance (NMR) and mass spectrometry (MS) are invaluable tools for structural insights. NMR provides information about the intricate molecular structure, including the arrangement of glucose units, side chains, and branching patterns within β-glucans. Mass spectrometry can elucidate molecular weights and reveal the specific structures in complex β-glucan mixtures. Immunological methods, such as enzyme-linked immunosorbent assays (ELISAs), leverage the specificity of antibodies to quantitatively measure β-glucan content, making them essential for applications in pharmaceuticals and biotechnology. Colorimetric and fluorometric methods employ reagents that change color or emit fluorescence upon interaction with β-glucans, providing rapid and high-throughput screening capabilities. Lectin-based assays use proteins that bind specifically to carbohydrates, allowing for sensitive qualitative assessments. Molecular techniques, including polymerase chain reaction (PCR), can identify and quantify β-glucan content by targeting genetic markers associated with β-glucan synthesis. These methods are particularly useful when working with microorganisms that produce β-glucans. Additionally, microscopic techniques like light microscopy, SEM, and TEM allow for visualizing the physical properties and arrangement of β-glucans in samples, which is essential in research and quality control. Biosensors are emerging as powerful tools for real-time, on-site measurement of β-glucan levels, particularly in food and pharmaceutical industries. These sensors can provide rapid and accurate results, enhancing quality control processes [33]. Figure 2 demonstrates an overview of the methods for screening β-glucans.

## 5. Extraction and Recovery of Beta-Glucan

In yeasts, the content of β-glucan lies between 5% and 7% [34]. *S. cerevisiae* has more than one-half of its cell wall composed of β-glucan [35]. Structurally, it is organized in the cell wall as linear with β-(1 → 3) linked D-glucan forming the backbone and β-(1 → 6) D-glucan forming the side chain branches [36]. The former has ~1500 residues and constitutes 85% of the β-glucan, while the latter makes up the remaining 15% with ~140 residues [37]. Another key component is the mannoproteins occupying ~35–40% of the cell wall. α-Glucan, proteins, chitin, and lipids are present in minor amounts. These macromolecules are interconnected through covalent bonds and provide cells with structural rigidity, resistance towards osmotic pressure, and other forms of environmental stress [38,39]. This, in turn, makes the recovery and extraction of β-glucan from the rest of the cellular components a multi-step process.

Apart from its extraction from the most widely exploited *S. cerevisiae* of the winemaking, brewing, bakery, and nutraceutical industries [40,41], attempts have been made to obtain and characterize the polysaccharide from other yeasts. A low-molecular-weight glucan with (1–6)-branched (1–3)-β-D-glucan was obtained by [42] at a high purity of 90 and 91.7% from a marine yeast *Debaryomyces hansenii* BCS004. Bzducha-Wróbel et al. (2015) [43] improved the β-glucan content in *Candida utilis* by using deproteinated potato juice water and glycerol as a carbon source to culture the yeast. By optimizing the culture conditions through changes in the nitrogen and carbon source, temperature, pH, and aeration, the β-glucan content in the cell wall increased to ~44–45%, a value significantly higher than when cultured in synthetic YPD media (~31%). A yeast strain of *Kluyveromyces marxianus*, TISTR 5925, was used by Vaithanomsat et al. (2022a) [44] and recovered 35.45% of β-glucan. Ref. [45] inspected the polysaccharide content and the yield % of alkali-soluble and alkali-insoluble glucan in strains of *Kloeckera apiculata*, *Debaryomyces hansenii*, *Zygosaccharomyces bailii*, *Kluyveromyces marxianus*, and *S. cerevisiae*. The alkali-soluble glucan varied between 10% and 48%, and the alkali-insoluble glucan was between 15% and 48%, depending on the strain.

Since the β-glucan in yeast is concentrated in the cell wall, having the cell wall free from other cellular content improves the efficiency of the subsequent steps [34,39]. The general methodology taken in its isolation proceeds by first carrying out a pretreatment step, followed by its extraction in the crude form and its final recovery in the pure form. The pretreatment step makes the extraction process easier by loosening and making the rigid cell wall porous. This increased permeability makes the cells more prone to external stress in the extraction step, which involves treatment with hot water, alkali, acids, enzymes, chemicals, solvents, ultrasound, and microwaves [46,47]. The release of cellular contents from the cell wall also aids in the extraction process through autolysis. In this process, when the cells are dying, the lytic enzymes become activated [48]. Its release from the vacuole and lysosome breaks down other membrane-bound organelles in the cell. The released glucanase and proteinase disorganize and crumple the cell wall to make it even more porous to completely release the intracellular contents into the surrounding medium [49]. Generally, the cell wall is separated from the cell-free extract by centrifugation and filtration. The β-glucan is finally purified from the crude extract by precipitation, ultrafiltration, or chromatographic techniques [50].

### 5.1. Pretreatment of Yeast Biomass to Enhance Extraction of Beta-Glucan

Generally, an initial pretreatment step is taken to permeabilize the cell wall. However, there is often very little or no clear distinction between the pretreatment and extraction steps because of their concomitant action on the cell wall and the intracellular material [51].

### 5.2. Mechanical Methods

Apart from the mechanical methods of cell disruption, which mainly affect the cell wall, the non-mechanical processes, because of their mode of action simultaneously on the cell wall and cytosolic content, are often considered both a pretreatment and extraction step. Shear forces are used in bead milling, high-pressure homogenizers, and ultrasonication, the mechanical technique used for yeast cell rupture. It mainly aims to disrupt the cell wall, allowing the intracellular contents to permeate.

#### 5.2.1. Bead Milling/Bead-Assisted Extraction

Although a non-selective method of cell disruption, bead milling has high efficiency in a single run, is easy to scale up because of its simple design, and has less labor requirements, giving a good extraction yield when coupled with different β-glucan extraction strategies. This pretreatment method is, therefore, a preferable one in the industry. The use of 0.5 mm zirconium-glass beads for homogenization of *S. cerevisiae* helped Bzducha-Wróbel et al. (2014) [52] achieve a high proportion of cytosolic content (~64–67%). The cell wall preparation was further analyzed, yielding 13–14% of β (1,3)/(1,6)-glucans when the milling process was carried out for 30 min. Marinescu and Stoicescu (2009) achieved a β-glucan content of 38.1% by only ball milling on spent brewer’s yeast for 20 min with five 12 mm diameter stainless-steel balls at 30 Hz. In another study, Dallies et al. (1998) [53] subjected a strain of *S. cerevisiae* to 0.5 g glass beads for four cycles, with each cycle being 20 s. The step was followed by different hydrolytic procedures to analyze different carbohydrate units in the yeast cell wall polysaccharide. Due to the prolonged time requirement in the lysis of spent brewer’s yeast cell wall when working on a small scale, Avramia and Amariei (2022) [54] optimized the number of 10 min vortex cycles, yeast to glass bead ratio, and yeast suspension concentration to increase cell disruption efficiency. Upon optimization, the β-glucan content extracted was 78.53%. Several parameters of the instrument operation determine the bead milling process of cell disruption. Important ones affecting the degree of disruption are glass bead concentration, rotational frequency, impeller geometry, operation temperature, rate of fluid flow, rheological properties of the cell suspension determined by cell concentration, and residence time [55]. Since the degree of cellular fragmentation determines post-processing and recovery, several studies have attempted to optimize these parameters and even draw a kinetic relationship between cell disruption and the release of cytosolic content.

#### 5.2.2. High-Pressure Homogenization-Based Extraction

Another mechanical cell disruption technology employed is high-pressure homogenization, wherein cells are subjected to high fluid shear forces while passing through a narrow channel at high pressure. The cell suspension is released into a low-pressure chamber with a cell wall disrupted fraction. Factors like applied pressure, nozzle diameter, temperature, biomass concentration, and number of cycles of passage through the channel control the disruption efficiency [56,57]. An exponential increase in the release of cellular content in *S. cerevisiae*, estimated through the cell disintegration index, was shown when Dimopoulos et al. (2020) [56] treated cell suspensions using a homogenizer. While at 100 bar pressure and eight passes, there was not much release of intracellular content; with an increase in pressure beyond 100 bar and the number of cycles, the cell disintegration index shot up rapidly. A similar effect of increased pressure on the release of higher amounts of enzymes, proteins, and other intracellular content was also observed in the studies carried out by Follows et al. (1971), Moore et al. (1990), and Spiden et al. (2013) [58,59,60]. Although in the process that was developed by Dimopoulos et al. (2020) [56] and Spiden et al. (2013) [60] high-pressure homogenization was used as a pretreatment step to extract β-glucan, X. Y. Liu et al. (2008) [61] and Thammakiti et al. (2004) employed the technique at a much later post-processing step [62]. When applied to autolyzed cells, Thammakiti et al. (2004) achieved improved β-glucan content [62]. Similarly, X. Y. Liu et al. (2008) also reported easier breaking of yeast cells and improved β-glucan amount when autolyzed and hot-water-treated cells were homogenized [61]. In general, treating cells under a high-pressure homogenizer before autolysis reduces autolysis time significantly compared to autolysis on non-homogenized cells.

#### 5.2.3. Ultrasonication-Based Extraction

A commonly used mechanical cell disruption technology on a lab scale is ultrasonication. Similar to the other two mechanical methods, the same principle of shear force causes cell rupture but in a much different way. Introducing high-frequency ultrasonic waves leads to cavitation at the nucleation sites of the sample or liquid close to the cells. Implosive collapse of cavitation bubbles close to such nucleation sites generates local areas of shockwaves, turbulence, and microjets, resulting in cell wall breakdown [63]. However, this process is also associated with generating high temperatures, pressures several times that of atmospheric pressure, and the free radicals OH, H_2_O_2_, and O, and cell structure damage through erosion and pitting, which can even lead to complete cellular disintegration, is necessary to optimize the operation parameters to reduce post-processing recovery and purification steps [64,65,66]. Critical parameters worth considering are power output, duty cycle, temperature, external pressure, depth of sono-reactor probe in the fluid, sonic waves (frequency, amplitude, and intensity), and lastly, the cell suspension’s pH, concentration, and ionic strength. Zhang et al. (2014) [66] conducted a study mainly focused on determining the effect of several factors that affect the effectiveness and selectivity of ultrasound-assisted extraction for either polysaccharides concentrated in the yeast cell wall or proteins in and within the boundary of the yeast cell membrane. A T/P factor representing the ratio of total polysaccharides to protein released under the action of ultrasonicator was taken as a measure of selectivity. The effect of pH, sonication intensity, yeast concentration, liquid volume used for processing, probe depth, ionic strength of the sample, and the concentration effect of left-over ethanol on spent brewer’s yeast showed similar selectivity on the release of polysaccharides and protein. However, temperature profoundly affected the selective release of polysaccharides over protein, with early release of polysaccharides followed by protein. Yuan et al. (2022) [67] observed structural modifications in the 3D structure of β-glucan extracted from the yeast *Candida utilis* while carrying out ultrasound enzymolysis. However, the modification due to the loss of the triple-helix structure of β-glucan improved its antioxidant properties and stability in an aqueous solution. The change increased its solubility in aqueous solution by lowering its thermal stability. A similar observation with increased solubility when ultrasonicated cells were treated with enzymes was also achieved by Zheng et al. (2019) [68]. Disruption of yeast aggregates caused by ultrasonication reduced its size from 8.80 μm to 1.77 μm, thereby improving the action of enzymes due to the better accessibility of active sites. The increased solubility, in turn, increased the yield of water-soluble β-glucan to 32.3% after enzyme treatment at 50 °C. However, Eom et al. (2021) reported no change in the overall chemical structure of β-glucan in the ultrasound-assisted yeast glucan extract [51]. The optimized operation conditions of 20 kHz and 800 W increased the β-glucan yield to 7.1% in 73.6% of the total extract.

### 5.3. Non-Mechanical Methods

Although mechanical methods demand lesser operational costs, easy implementation at the industrial scale due to simplicity in scale-up, and large-scale cell disruption, the energy requirements are high, and non-selectivity in cell disruption makes different non-mechanical methods, like physical and chemical, enzymatic, and electrical methods, the preferred choice. To understand the reason behind the selectivity of non-mechanical methods, it is necessary to have a clear understanding of the difference between cell disruption and cell fragmentation. Cell disruption is used in a broader sense to refer to the extent of wall rupture in a single cell and the proportion of cells in the entire population having at least some rupture in their cell wall. Fragmentation, conversely, is a special case of a higher amount of cell disruption that has caused intact cells to be broken into small particles [60]. This issue is commonly seen in mechanical methods of cell rupture, in which case it is challenging to separate fine-sized fragmented cells from all the other materials inside the cells because of their presence. The result is a drop in the overall yield of β-glucan from lost cell walls and the intracellular content. To eliminate occurrences of this nature, selective methods like treatment with chemicals, enzymes, and applied electric fields are adopted to cause cell disruption. The selectivity is achieved through controlled disruption or cell permeabilization. The use of specific solvents and surfactants in the chemical method or exposure of cells to the electric field in the electroporation method permeabilizes the cell wall to allow exudation of the inner cell material through the pores. Leaving the cell wall free from the rest of the cellular material can serve as a benefit to separate the β-glucan from the only place where it is concentrated by centrifugation [69]. However, the challenge is maintaining the overall integrity of the β-glucan and its associated activity. More or less, the associated structural changes brought about due to such treatment result in some changes or loss in inherent β-glucan functionality [70]. Also, in some cases, the presence of chemicals and enzymes adds to additional steps and costs in the pure-form recovery of β-glucan. Scale-up is another issue that needs to be considered when considering its adoption at the industrial level.

#### 5.3.1. Pulsed Electric Field-Based Extraction

Pulsed electric field-based extraction is a non-thermal mode of cell wall permeabilization induced by a high DC voltage (kV pulses) in a pulse or intermittent manner. Like electroporation, which is commonly used to disrupt cell membranes, this method is equally effective in the case of cell walls. When the aqueous cell suspension is exposed to such an effect, most of the cell walls become porous either reversibly or irreversibly. As the porosity increases, more intracellular material emerges into the media [71]. Prior knowledge of the cell wall thickness, rigidity, and structure/shape therefore proves beneficial in fine-tuning the operating parameters of the process [72]. The factors that need to be worked on for optimal extraction of intracellular proteins and other materials include electric field intensity, temperature of the aqueous solution, cell concentration, duration of operation, pulse waveform, and voltage [73]. To increase the efficiency of cell autolysis, Dimopoulos et al. (2018) [74] exposed a yeast cell suspension to a range of electric fields from 5–20 kV/cm and used the cell disintegration index to assess the efficiency in the release of yeast extract. In a recent study, Berzosa et al. (2023) [75] devised a strategy to separate yeast biomass into three fractions to isolate industrially important products from each. The group successfully obtained a β-glucan pellet in the final fraction separated from the rest of the cellular components. They also demonstrated how the intensity of the pulsed electric field could be tuned to enrich the components in each fraction.

#### 5.3.2. Enzyme-Assisted Extraction

A well-targeted method of cell disruption is via the use of enzymes. Both commercially available enzymes, like mannase, protease, chitinase, lipase, glucanase, glucosidase, and cellulases, and microbial enzymes can be employed to achieve enzymolysis. At an affordable price, commercial enzymes such as glycosidase, peptidase, glucanase, zymolyase, lysozyme, and lipase have been reported to be most effective in the cell wall lysis of yeasts [76]. In a specific type of cellular treatment, the endogenous enzymes of the yeasts can also be activated through autolysis to target cell lysis from the inside. Because of enzymes’ inherent property to show their activity at a mild temperature, pH, and specific action on the target, the process has an added selectivity and low-energy operational advantage [77]. However, applying the correct set of enzymatic combinations is crucial in making the cell wall porous to produce the desired component in the highest proportion in the isolated fraction. To target cell rupture solely by enzymatic method, the aim is first to disorganize β-1,6-glucan, the main strength-providing structural component. It cross-links with β-1,3-glucan and covalently binds to the cell wall mannoproteins via the β-1,6-glucosidic bond [78]. Initially, protease and glucanase are generally used to cause a collapse of the tight bindings on the other cell wall components [79,80]. To ensure that the main structure of β-glucan remains undamaged, a vital optimization factor is the concentration and the duration of the enzymes’ action on the cell wall components. On the other hand, protease loosens the proteins’ hold on mannans in the mannoproteins, causing their release. Subsequently, when the desired product is β-glucan, the action of lipases, chitinases, and proteases is kept active while deactivating the initial activity of glucanase to keep the compound of interest in its non-hydrolyzed form [81]. Once the cell wall becomes weak, osmotic cell lysis is promoted by the addition of inorganic salts (e.g., sodium chloride), organic solvents (e.g., ethanol and toluene), or ethyl acetate [49,76]. Javmen et al. (2012) used a naturally present microbial enzyme complex from the yeast *Actinomyces rutgersensis* to achieve lysis of *S. cerevisiae* to extract β-glucan [82]. The isolated enzyme complex was a cocktail of several lytic enzymes, with β-glucanase, mannanase, chitinase, and proteinase being the important ones worth mentioning. Under the coupled action of lytic activity from the components of the enzyme complex, cell lysis was much faster than the autolysis method, which takes a few days to complete, resulting in increased chances of contamination. Finally, the β-glucan was isolated from the insoluble cell wall fraction by applying mild alkali treatment. The optimal concentration of the lytic enzyme Glucanex^®^ 200G for β-glucan extraction was determined by Varelas, Tataridis et al. (2016) using three different enzyme concentrations [37]. The maximum content was obtained when a concentration ten times higher than the normal concentration (10 × 0.015 g/L) was used. For the extraction of β-glucan, Borchani et al. (2014) focused on eliminating the protein and lipid fraction to produce a concentrated glucan fraction at the end of the enzyme treatment process [83]. The suspension of the yeast cell wall was subjected to commercial-grade protease (Savinase^®^) and lipase (Lipolase^®^) enzymes to create a β-glucan yield of 18% (*w*/*w*) of the original ratio in the yeast cell walls with a purity of 79%. As the entire process left the chain undegraded, it preserved the glucan structure native to the cell wall. Vaithanomsat et al. (2022b) assessed the effect of treatment of the protease enzyme Alcalase^®^ on *Kluyveromyces marxianus* for the recovery of β-glucan [44]. In total, 81.15% recovery of β-glucan was obtained upon enzyme hydrolysis at 55 °C for 5 h. However, the purity tested through FT-IR and NMR spectroscopy was low due to the serine endopeptidase’s random hydrolysis of internal peptide bonds. A spent brewer’s yeast hydrolysate as value-added protein-rich content was derived from spent brewer’s yeast when Marson et al. (2019) carried out hydrolysis using the proteolytic enzymes Brauzyn^®^, Alcalase™, Protamex™, and Flavourzyme™ [84]. A preceding disruption method often enhances enzymolysis due to increased contact surfaces and loosening of β-glucan aggregates, thereby providing more active sites for enzyme action. The application of ultrasound and alkali as a pretreatment for enzymatic hydrolysis increased the yield of water-soluble β-glucan to 32.3% in the case of ultrasound-coupled enzymolysis and 36.2% in the case of alkali-assisted enzymolysis. Reduction in yeast glucan size in both the pretreatment methods was attributed to enhanced hydrolysis and water solubility [68].

#### 5.3.3. Alkaline Extraction

β-glucans are more prone to isolation from disintegrated yeast cell walls using alkali rather than intact cells. Sodium hydroxide is typically used; however, potassium hydroxide can also be used [85]. A novel approach of isolating β-glucan was carried out by autolyzing winery spent yeast biomass and extraction with sodium hydroxide, which resulted in 43.37% *w*/*w* of β-glucan from white wine lees and 28.95% *w*/*w* from red wine lees [37]. The alkaline extraction of *S. carlsbergensis* strain isolated from beer beverage waste resulted in an 8.43% *w*/*w* of β-glucan with a diammonium sulfate medium (MDS), which was enhanced to 11.99% *w*/*w* from an MDS medium supplemented with 0.1% *w*/*v* tannic acid [86]. A 2% *w*/*v* NaOH treatment at 90 °C for 5 h followed by neutralization with sodium acetate, with hot water extraction, was able to remove the cellular proteins and improve their solubility [83,87,88]. Alkali treatment at high pressure improved the extraction efficiency, producing 78.11% pure β-D-glucan while reducing the processing time and the alkali dosage [89]. At the same time, a lower concentration of NaOH was more efficient in extracting β-glucan (65.366% with 1 M NaOH and between 80 and 90% with 0.5 M NaOH) than higher concentrations. Higher temperatures (63.793% and 95% yield at 90 °C) and neutral pH (65.25% yield at pH 7) are also favorable [12,82,90].

#### 5.3.4. Acidic Extraction

While β-glucans are generally insoluble, acid degradation with 45% H_2_SO_4_ was used to solubilize β-glucans into low molecular weights with higher purity. Acid hydrolysis with 12 M sulfuric acid, sodium acetate treatment, and enzymatic hydrolysis was also used to determine β-glucan content [91].

### 5.4. Purification and Characterization

Since yeast cell walls also contain lipids and proteins, these must be separated once the extraction process is complete. Centrifugation techniques and column chromatography methods, such as ion exchange and size-exclusion chromatography, are famous for solid–liquid separation [88]. Once purification is achieved, it is recommended to use dynamic light scattering (DLS), Fourier transform infrared spectroscopy (FT-IR), or nuclear magnetic resonance (NMR) for structure confirmation and characterization of β-glucan [12,44,90,92]. Table 1 lists the various extraction methods and their respective advantages or disadvantages.

## 6. Health Benefits of Beta-Glucans

### 6.1. Immunomodulatory Effects

Yeast (1,3)-β-glucan stimulates the synthesis of cytokinin, interleukin, and antibodies by immune cells such as macrophages and neutrophils. This stimulation primes the body to fight disease more effectively, preserving lymphocytes’ ability to make cytokines. It also increases IgM antibodies, boosts humoral immunity, and stimulates TLR2, NF-B activation, and TNF-secretion. It also boosts the immunity of the mucous membrane in the digestive tract, allowing it to protect against bacteria and other substances. Yeast glucan binds to macrophage, neutrophil, and lymphocyte surface receptors, altering cellular signaling and allowing lymphocytes to access infection sites. It functions like an immune response stimulant and promotes immune cell activation, boosting the immune response cooperatively [93].

(1 → 3)-β-glucan is an immunomodulator that increases the anti-tumor activity of peritoneal macrophages. A specific activation mechanism triggers T cells, macrophages, natural killer cells, and complement components. When present, it combines to complement component C3, activating the C3 convertase enzyme and breaking down C3 into iC3b, signaling immune cells to destroy invaders [93].

Zheng et al. examined the immunomodulatory implications of five different water-soluble yeast glucan fractions (WYG1-5) in RAW264.7 macrophages. According to the findings, WYG boosted nitric oxide generation, phagocytosis action, and cytokine secretion. NO generation, phagocytosis, and cytokine release were all reduced by WYG and lipopolysaccharide incubation. WYG’s structure–activity relationship suggests a pharmacological basis for managing inflammatory diseases [94].

Van Steenwijk et al. emphasized the possible use of fungal β-glucans, immunomodulators in traditional Chinese medicine, nutrition, and medicine. These glucans can potentially be employed in pharmaceuticals and foods to promote immune responses. The consequences could be medicinal or beneficial to one’s health [95].

### 6.2. Anti-Tumor Properties

Sun et al. discovered that giving pure baker’s yeast polysaccharides to colitis mice lowers IL-6, iNOS, IL-1, macrophage invasion, and colonic mucosal damage. According to Geller et al., yeast-derived β-glucans modify cancer therapy’s tumor milieu and immune-suppressive cell phenotype by activating transcription factors such as NF-B [96].

Wang et al. (2020) identified an anti-tumor mechanism in water-soluble yeast glucan, suggesting its potential in the treatment of liver cancer. The polysaccharide hindered autophagic breakdown by elevating lysosomal pH and lowering lysosomal cathepsin activity [97]. It successfully suppressed tumor formation in primary HCC models and xenografts while causing no harm. β-glucan exerted direct anti-tumor effects in immune-deficient BALB/c nude mice without relying on the immune system [98].

### 6.3. Antioxidant Activity

Zhang et al. researched the in vitro antioxidant activity of β-glucan and observed significant clearance effects on OH, DPPH, and ABTS, with ABTS showing the highest efficacy. Consequently, this study proposed a hypothesis suggesting that the Area gene governs the synthesis of β-glucan with antioxidant properties [99].

According to Kofuji et al., β-glucan derived from barley has high antioxidant activity, regulated by its physiological characteristics and extraction process. Its hydroxyl scavenging action is more significant than other food additives, implying that it could be used as a dietary supplement and food alternative for improved fitness and beauty [100].

Although yeast glucans have biological properties such as oxidation inhibition, its low water solubility restricts its applicability. H_2_O_2_ treatment with ultrasound improves solubility and antioxidant activity [101].

Extracts of spent brewer’s yeast (*Saccharomyces pastorianus*) are high in RNA, minerals, essential amino acids, and vitamins, thus rendering them suitable for functional foods and nutraceuticals. Vieira et al. investigated these extracts’ nutritional makeup, antioxidant properties, and phenolic components, emphasizing their potential as a low-cost and fascinating food or nutraceutical supplement. Using lyophilized brewer’s wasted yeast extract for β-glucans and fiber improves the application of yeast cell walls for fiber and β-glucans, making it an intriguing and beneficial nutraceutical ingredient [102].

### 6.4. Cholesterol-Lowering Effect

Elevated cholesterol levels have been related to an increased risk of cardiovascular disease, making the hunt for an organic cholesterol regulator critical. Dietary fiber, which Keys et al. first proposed, was later discovered to have actions relating to glucans, a collection of biological response stabilizers. β-glucans are β-glucose polymers [103,104].

Researchers discovered that feeding yeast-derived β-glucan to mice resulted in a dose-dependent drop in plasma cholesterol levels, with insoluble Betamune exhibiting a more significant effect. The results were dose-dependent and differed depending on the type of glucan used. Macrophages digested both insoluble and soluble glucans, altering their biological activity. The results also validated the macrophage–cholesterol axis concept, implying that these glucans can alter plasma cholesterol levels [104].

*S. cerevisiae* includes β-glucan, a cell wall component that improves immunological function. In hypercholesterolemic patients, soluble glucans can reduce the concentration of low-density lipoprotein and cholesterol while strengthening the immune system and lowering cholesterol levels. Antiseptic, antioxidant, anti-inflammatory, immune system activators, shielding against radiation, and cholesterol-lowering properties are among the many advantages of glucans.

Sprague Dawley rats were used to assess the potential utility of β-glucan extract as an anticholesterol drug. The results demonstrated that a 10 mg dose of *S. cerevisiae* β-glucan extract could lower total cholesterol to levels close to normal at 32.79% (blood plasma) and 33.71% (liver) [105].

### 6.5. Diabetes Management

Fungal β-glucans have been shown in animal tests and clinical trials to lower blood glucose levels following oral treatment. Orally consumed fruiting bodies and acidic polysaccharides from *Tremella mesenterica* and *T. aurantia* lower blood glucose levels in diabetic mice. In diabetic rats, β-glucans derived from *Agaricus blazei* basidiocarps exhibit antihyperglycemic, antihypertriglyceridemic, antihypercholesterolemic, and antiarteriosclerotic activity [106].

Type 2 diabetes, as well as obesity, is linked to changes in the gut flora. By influencing glucose, cholesterol, and lipid homeostasis, yeast glucans have the ability to modulate the innate immune-metabolic response. A study examined how yeast glucan interacted with an obese normal or obese diabetic intestinal microbiome to influence metabolic health via hepatic effects during high-fat food stress. Male C57BL/6J mice fed a high-fat diet and pre-inoculated with the intestinal microbiota coming from obese normal or obese type 2 diabetic patients demonstrate a poor metabolic state and resistance to insulin. Yeast glucan supplementation fixes these concerns by boosting the quantity of health-related microorganisms [107].

The ability of beta-glucan to produce sticky solutions is useful because the more significant the viscosity of the phase, the lower the glucose and insulin levels in blood plasma, and this contributes to its effectiveness in decreasing blood glucose, but it also relies upon its concentration and molecular weight [108].

### 6.6. Anti-Inflammatory Effects

Yeast glucans are obtained from the cell walls of yeast and possess various biological activities, including anti-inflammatory, anti-tumor, and immunomodulatory properties. However, their insolubility in water restricts their potential applications in the biomedical and food industries. Alterations and increases in β-(1,3)-glucans’ water solubility could broaden their scope. Earlier studies have demonstrated that yeast β-glucans lower intestinal inflammation [109,110].

The use of alternative feed additives, such as yeast β-glucans, has been proven to be safe and potentially reduces the immunological response to inflammatory stress in egg-laying hens and birds in normal conditions. Yeast β-glucans may be partially responsible for this, as they regulate the constitution of gut microbes by increasing the number of beneficial microbes in the gut and repressing the growth of pathogens. The present research results support the idea that nutritional therapies could be employed to relieve immunological and inflammatory stress responses in laying hens without affecting production efficiency [111].

**Figure 3:**
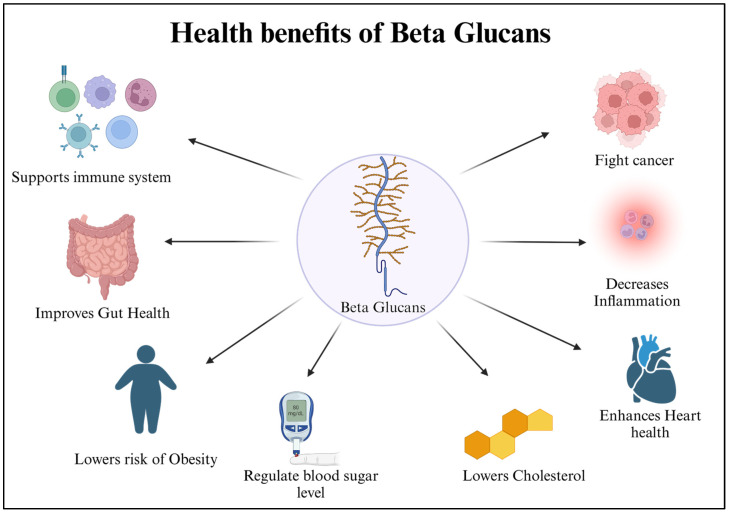
Health benefits of β-glucans. β-glucans help support the immune system and decrease inflammation. They improve gut and heart health. They regulate blood sugar and cholesterol levels, helping to lower the risk of obesity. They might also help fight cancer.

Despite in vitro investigations, various animal experiments have been undertaken to assess β-glucans’ anti-inflammatory properties. Carbohydrate anti-inflammatory studies have yielded excellent results. The anti-inflammatory properties of lentinan in the intestine were investigated. To investigate the effects of lentinan in vivo, colitis mice administered dextran sulfate sodium (DSS) were employed. Only a handful of studies involving humans have looked at the impact of β-glucans on inflammatory processes throughout the body [112]. Figure 3 illustrates the different health benefits of β-glucans.

## 7. Applications for Beta-Glucans

Yeast-extracted β-glucans, also known as yeast β-glucans, have various applications across various industries due to their unique properties and potential health benefits. Here are some key applications of yeast-extracted β-glucans.

### 7.1. Functional Foods and Beverages

The food industry is exploring innovative and healthy ingredients to improve the nutritional and functional qualities of products while also reducing production costs. Yeast β-glucans were deemed safe for consumption by the European Food Safety Authority in 2011. Moreover, the United States Food and Drug Administration has granted them the Generally Recognized as Safe (GRAS) status (GRN No. 239 Bakers Yeast Beta-Glucan, 2008). Indeed, yeast β-glucans have been incorporated into various food products, and they are commercially available for food supplementation, with examples such as Wellmune^®^, Goldcell^®^, and Yestimun^®^.

Yeast β-glucans can serve as ingredients and enhancers for various foods and beverages, improving their characteristics. They can substitute for components such as fat, impacting the nutritional, technological, and sensory properties. Additionally, they contribute to thickening, emulsification, stabilization, and gelation in a range of food products, including bakery, meat, and dairy items [113]. Furthermore, β-glucans act as water retainers and adequate fat replacements, delivering a pleasing mouthfeel like fat [62]. Even in small concentrations, they can alter sensory properties, viscosity, and rheology.

In the dairy industry, β-glucans exhibit significant technological potential by boosting overrun in ice cream, binding free moisture, serving as substitutes for milk fat, aiding in structure formation, and improving cheese yield, even at low concentrations (0.5–3.0%). Furthermore, using BSY β-glucan as a fat replacer in skim milk yogurt has enhanced rheological properties and physical stability.

In bakery items, β-glucans can modify the expansion rate, decrease volume, elevate bread firmness, and influence the gluten matrix, water absorption, and pore formation. Furthermore, the supplementation of BSY β-glucan in bread formulations improves the nutritional and health-promoting characteristics of the product while extending its shelf life during refrigerated storage [114,115]. Hence, it is essential to evaluate the implications of adding β-glucan and identify the optimal concentration to mitigate its effects on the product, considering its biological properties [116].

In meat products, β-glucan can be a substitute for reducing fat, reducing cooking losses in emulsions, enhancing viscosity, and preserving moisture and fat by forming a three-dimensional network. This leads to meat products with a softer texture [117].

BSY β-glucans can be integrated into mayonnaises as a substitute for fat, improving storage stability and reducing calorie content [118]. β-glucans also provide protective benefits for lactobacilli in freeze drying, refrigerated storage, and exposure to simulated gastrointestinal conditions. This makes them valuable additions to functional foods containing probiotics [119].

### 7.2. Nutraceuticals and Dietary Supplements

β-glucans are commonly used as supplements in the form of capsules, tablets, or powders for their immune-boosting properties and potential to support overall health. They are often taken as dietary supplements to enhance immune function and promote general well-being.

Yeast-extracted β-glucans have several applications in nutraceuticals and dietary supplements. They are commonly used as an ingredient in these products due to their potential health benefits.

In nutraceuticals, yeast-extracted β-glucans are used to support immune health. They have immune-enhancing properties and can stimulate the immune system, helping to modulate immune responses. This can be beneficial in promoting a healthy immune system and protecting against infections. β-glucan derived from brewer’s yeast offers numerous biological benefits, including antibacterial and antioxidant properties. It serves as a food additive and dietary supplement to aid digestion, strengthen the immune system, and help lower liver and blood lipid levels. Dietary supplements also utilize yeast-extracted β-glucans to provide additional nutrients and support overall health. These supplements may come in capsules, tablets, powders, or liquids. Yeast-extracted β-glucans in dietary supplements are commonly taken orally and can help support immune function, improve gut health, and potentially manage cholesterol levels. They can be combined with other beneficial ingredients to enhance their effectiveness [120].

### 7.3. Pharmaceutical Formulations

Yeast extracts find applications in the medical and healthcare domains owing to their biological activities, rich nutritional content, disease management and prevention capabilities, and their contribution to enhancing intestinal microbial balance [121]. Conventional anti-inflammatory and antibacterial therapies often incorporate yeast extract, and β-glucan derived from yeast cells provides comparable health advantages to β-glucan sourced from cereals. Yeast extract is applicable for addressing skin conditions, such as pruritus (itchy skin), a condition affecting approximately 13.5% of the global population [104,122]. Yeast extract can manage skin conditions like pruritus (itchy skin), which impacts roughly 13.5% of the global population. In comparing a yeast extract formulation and a traditional treatment, colloidal oatmeal lotion (CO), the former demonstrated high effectiveness and superiority over CO [123]. The effectiveness of yeast extracts is mainly ascribed to flavonoids, dextran, amino acids, and vitamins. These components have the capability to obstruct diverse histamine receptors, consequently inhibiting pro-inflammatory factors and alleviating itching [124,125].

In medical contexts, yeast extract is predominantly recognized for its anti-inflammatory properties, encompassing emphysema and pneumonia [126]. In a mouse model of cigarette smoking, the oral administration of yeast extract led to a significant decrease in pro-inflammatory cell numbers in lung alveoli. This reduction was accompanied by lower levels of inflammatory mediators COX-2 and NOS, attributed to the antioxidant and anti-inflammatory properties inherent in yeast extract [127]. Nevertheless, the research on using yeast extracts to mitigate pneumonia and other inflammatory lung conditions is still limited. Further investigation is warranted to delve into their potential in this regard.

Besides its anti-inflammatory and anti-cancer properties, oral β-glucan provides a range of health advantages, including reducing cholesterol and blood lipid levels, all without the side effects associated with synthetic drugs [128]. It additionally hinders the formation and maturation of adipocytes, primarily achieved through the suppression of adipogenic differentiation [104,122]. Obesity is closely associated with the regulatory factors governing adipocyte differentiation, implying that yeast β-glucan exhibits significant potential for advancing treatments for conditions such as obesity, pneumonia, cardiovascular disease, and skin diseases [129,130].

### 7.4. Cosmetic and Skincare Products

The cosmetics industry is extensive and lucrative, yet it often faces safety and efficacy challenges, demanding thorough testing when incorporating new ingredients into cosmetic products. Yeast extract is a well-established ingredient in cosmetics, containing amino acids, polysaccharides, polypeptides, proteins, and other compounds that deliver various beneficial effects when applied topically. These advantages include skin moisturization, cell renewal stimulation, slowing skin aging, and accelerating wound healing [87,131]. Usually, yeast extract is blended with additional vitamins, moisturizers, and antioxidants to fulfill specific cosmetic purposes [132]. An oral tablet incorporating vitamins and yeast extract has been created to address sunburn by diminishing cell damage and lipid peroxide levels in the skin. This underscores the potential for yeast extract to mitigate photo-aging and oxidative stress in the skin [133].

However, thorough safety testing is imperative for all new ingredients and products to ascertain the absence of adverse side effects. To assess potential skin irritation, yeast extract was combined with conventional skincare ingredients such as vitamins A, C, and E and applied to the skin of healthy individuals. After several days of consistent use, no skin irritation was identified for any formulation. Moreover, all formulations reduced skin roughness and increased the water content of the stratum corneum [134]. While these preliminary findings show promise, additional testing is necessary to validate the safety of such formulations.

Yeast extract has the capability to degrade and eliminate melanin from human skin, rendering it suitable for use in skin-lightening products. In comparison between a traditional skin lightener and one containing natural active ingredients such as yeast extract and salicylic acid, both formulations demonstrated equal effectiveness in reducing spot intensity and improving pigmentation. However, natural formulation offers the advantages of more abundant raw materials and lower production costs. Typically, formulations incorporating natural ingredients like yeast extract exhibit better long-term tolerability than those containing synthetic chemicals, indicating more significant potential for future development [132]. Notably, the utilization of yeast extract in skincare products is relatively recent and somewhat constrained, leaving ample room for the development of new applications and products in this field.

### 7.5. Animal Feed and Veterinary Applications

Yeast extracts are recognized as a valuable nutrient source in animal feed, explicitly emphasizing their application as an additive for poultry [135]. These extracts can be formulated to contain an abundance of polysaccharides like β-glucan, mannan [136], and chitin [137], making them well suited for poultry farming and aquaculture, where they play a role in enhancing the immune function of poultry [138,139].

The livestock industry is confronted with escalating challenges, encompassing the demand for accelerated animal growth, heightened animal health issues, and the emergence of antibiotic-resistant bacteria. Certain countries have prohibited using antibiotics and spray-dried plasma protein (SDPP) in animal feed to address these concerns. This has led to a growing interest in immune-boosting products, such as yeast extracts, as substitutes for antibiotics and SDPP [140]. The incorporation of yeast extract into the daily diet of turkeys has demonstrated improvements in their growth and immunity. This enhancement is accomplished by stimulating the oxidative burst activity of heterophilic cells and elevating parameters such as red blood cell count, uric acid levels, and blood hemoglobin content, among others [141]. Yeast extracts are potential substitutes for antibiotic-based animal growth enhancers [141,142]. When incorporated into fish feed, yeast extract enhances the immunity of fish species like Rosita, resulting in elevated levels of white blood cells, serum proteins, and globulins. It has demonstrated greater efficacy than alternatives such as brewer’s yeast or spirulina [140]. The active components accountable for these effects are β-glucan and mannan, which are antibacterial agents that regulate intestinal flora and stimulate immune system function. These benefits extend not only to animals but also to humans [143,144].

Yeast extract also functions as an unconventional yet significant protein source for animal feed [140]. A comparative study involving pig feed added protein supplements such as yeast extract and SDPP. Yeast extract exhibited superior performance in terms of ileal digestibility, amino acid digestibility, metabolizable energy, and apparent digestibility, outperforming SDPP [145]. Yeast extract is anticipated to emerge as a notable alternative for protein ingredients in poultry feed. Furthermore, when integrated with fish meal in varying proportions for shrimp feed, yeast extract resulted in heightened digestive protease activity and a more efficient feed conversion rate for the shrimp. This suggests that yeast extract can potentially replace a considerable portion of fish meal, significantly reducing feed costs [146]. In summary, yeast extract serves as an immunomodulator and nutritional supplement with the potential to replace conventional protein ingredients in animal feed.

### 7.6. Industrial and Biotechnological Uses

Yeast extract is an immensely valuable growth medium for microbial fermentation, applicable in laboratory and industrial environments. This is particularly true for auxotrophic strains, as yeast extract is rich in amino acids, vitamins, nucleosides, polypeptides, and minerals [147]. However, minor differences in the yeast source and production process can result in significant variations in the composition of the extract. Therefore, the meticulous selection of an extract customized to the particular microorganism of interest is crucial.

Among the diverse nutrients in yeast extract, polypeptides play a crucial role in influencing the growth and metabolism of fungi. Yeast extracts can encompass as many as 4000 oligopeptides [148]. Even small amounts of peptides can substantially impact the metabolic activity of bacteria during fermentation [149]. Peptides and amino acids in yeast extract are crucial elements in the growth of lactic acid bacteria, contributing to as much as 60% of the biomass [150]. Significantly, yeast extract has become the established growth medium to produce hyaluronic acid (HA) using the auxotrophic *Streptococcus zooepidemicus*. This choice is based on its adherence to the criteria of cost-effectiveness and ready availability while also supplying essential nutrients for the fermentation of *S. zooepidemicus* [151]. Moreover, yeast extract has been demonstrated to enhance the fermentation capabilities of *S. cerevisiae* in tequila production. This involves mitigating nutritional limitations and enhancing fermentation and aroma production [152]. Due to its characteristics, yeast β-glucan can substitute conventional ingredients employed on an industrial scale, including alginates, gum arabic, pectin, and carboxymethylcellulose, often providing comparable or enhanced properties [153].

Recent reports emphasize the favorable influence of yeast extract as a culture additive on the stability of *Acidithiobacillus ferrooxidans* in the presence of oil as a biological source. This inclusion decreases the leaching concentration of the highly toxic waste, arsenic (As), in acid water, reducing it from 13.78 mg/L to 7.23 mg/L. This enhancement leads to a 40% increase in stability and substantially contributes to the control of arsenic content [154].

Leveraging the nutrient-rich and safe properties of yeast extract [155] and harnessing the nutrient-rich and safe characteristics of yeast extract, innovative applications were explored in nanoparticle technology, leading to a novel approach for synthesizing antibacterial silver nanoparticles (AgNPs). These AgNPs maintain their antibacterial properties and are environmentally friendly, safe, and non-toxic, thereby paving the way for novel applications of yeast extract.

**Figure 4:**
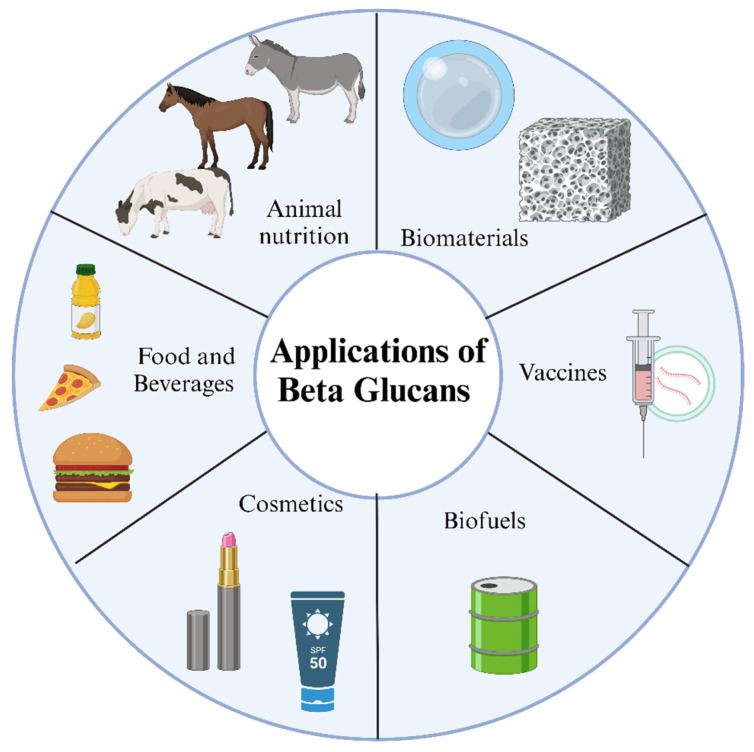
Applications for β-glucans. β-glucans have various applications, such as in animal nutrition, biomaterials, cosmetics, biofuels, vaccines, and food and beverages.

In summary, yeast extract is a cost-effective growth medium with an ideal nutrient composition for various fermentation applications. However, selecting an appropriate extract for each microorganism is essential to optimize the fermentation process, as the composition and quality of yeast extract can vary due to differences in raw materials and processing methods. Figure 4 illustrates the different applications of β-glucans.

## 8. Conclusions

In conclusion, this comprehensive review has provided a detailed exploration of the production, extraction, and applications of β-glucans produced by yeasts. We began with an overview of β-glucans, highlighting their importance in health and various industries. The pivotal role of yeast as a microorganism for β-glucan fermentation was discussed, encompassing yeast types, properties, and the selection of suitable strains. The fermentation process was thoroughly examined, from substrate selection to inoculum development and fermentation conditions. Furthermore, we delved into the methods for screening, extraction, and recovery of β-glucans, exploring both chemical and physical extraction techniques and solid–liquid separation and concentration methods. Purification techniques, such as filtration, centrifugation, precipitation, chromatography, and membrane separation, were elucidated to ensure the purity of the extracted β-glucans. This paper also highlighted the significant health benefits of β-glucans, including their immunomodulatory, anti-tumor, antioxidant, cholesterol-lowering, diabetes management, and anti-inflammatory effects. These findings underscore their potential as valuable components in various medical and health applications. Lastly, we discussed the diverse applications of β-glucans in functional foods, nutraceuticals, pharmaceutical formulations, skincare products, animal feed, and industrial biotechnological uses. Their versatility in different sectors demonstrates the wide-ranging impact of β-glucans on society and the economy. In summary, this review has provided an in-depth understanding of β-glucans and shed light on their remarkable potential in both health and industry. As research continues to evolve in this field, it is expected that β-glucans produced by yeasts will play a significant role in shaping the future of food, medicine, and biotechnology.

## Figures and Tables

**Table 1:** Advantages and Disadvantages of Different Methods of Beta-Glucan Extraction.

Method	Advantage	Disadvantage
Chemical Methods		
Alkaline Extraction	Efficient extraction of β-glucans; alkaline conditions enhance solubility.	It may cause degradation of some polysaccharides, requires careful pH control, and can be harsh on labile structures.
Acidic Extraction	Efficient extraction of β-glucans from fungal cell walls; cost-effective and straightforward process.	May lead to partial degradation of β-glucans due to acid hydrolysis; requires careful optimization to avoid undesired side effects.
Enzymatic Extraction	Enzymatic extraction of β-glucans is efficient, specific, and yields high purity, avoiding harsh chemical treatments.	It may be costlier than chemicals, and enzyme availability and specificity can vary, influencing extraction efficiency.
Physical Extraction Methods		
Hot Water Extraction	Hot water extraction of β-glucans is a simple, cost-effective method that retains bioactivity and is environmentally friendly.	High temperatures may cause degradation of β-glucans, and the extraction process may not yield high purity compared to other methods.
Ultrasonic Extraction	Ultrasonic extraction of β-glucans is rapid and efficient, providing high yields quickly.	The potential for sample heating, degradation, and equipment costs may be higher than traditional methods.
Microwave-Assisted Extraction	Microwave-assisted extraction of β-glucans offers rapid extraction, reduced solvent usage, and enhanced yields compared to conventional methods.	Potential sample degradation due to high temperatures and equipment costs may limit accessibility for some laboratories.
Solid–Liquid Separation		
Centrifugation	Efficient separation of β-glucans from other components, providing high purity in a relatively short processing time.	May require specialized equipment, and the high force involved could affect the structural integrity of β-glucans.
Filtration	Filtration extraction of β-glucans allows for a selective isolation process, yielding a purified product with reduced impurities.	It may require specialized equipment, and the process could be time-consuming, potentially limiting its scalability for large-scale production.
Sedimentation	Efficient method for isolating β-glucans from natural sources, providing a relatively simple and cost-effective process.	This may result in variable purity, which might be time-consuming compared to some modern extraction techniques.
Concentration Methods		
Evaporation	An efficient extraction method for β-glucans, concentrating the target compound by evaporation.	May be time-consuming, and some heat-sensitive compounds could degrade during evaporation.
Spray Drying	Efficient method for extracting β-glucans from various sources, producing a dry and easily transportable product.	This may lead to the degradation of heat-sensitive compounds, and the process can be energy-intensive.
Freeze Drying	Preserves the structural integrity and bioactivity of β-glucans, providing a high-quality product with extended shelf life.	It requires specialized equipment, is time-consuming, and may result in higher production costs than alternative extraction methods.

## Data Availability

All the information was taken from several research papers and review papers for our review work.
